# Sensitization profiles to house dust mite *Dermatophagoides pteronyssinus* molecular allergens in the Lithuanian population: Understanding allergic sensitization patterns

**DOI:** 10.1002/clt2.12332

**Published:** 2024-01-24

**Authors:** Gabija Biliute, Monika Miskinyte, Asta Miskiniene, Aukse Zinkeviciene, Violeta Kvedariene

**Affiliations:** ^1^ Faculty of Medicine Clinic of Chest Diseases Allergology and Immunology Institute of Clinical Medicine Vilnius University Vilnius Lithuania; ^2^ Faculty of Medicine Vilnius University Vilnius Lithuania; ^3^ State Research Institute Centre for Innovative Medicine Department of Immunology Vilnius University Vilnius Lithuania; ^4^ Department of Pathology Faculty of Medicine Institute of Biomedical Sciences Vilnius University Vilnius Lithuania

**Keywords:** component‐resolved diagnostics, cross‐reactivity, house dust mite allergy, molecular Allergology, sensitization patterns

## Abstract

**Background:**

House dust mite (HDM) allergy is a prevalent global health concern, with varying sensitization profiles observed across populations. We aimed to provide a comprehensive assessment of molecular allergen sensitization patterns in the Lithuanian population, with a focus on *Dermatophagoides pteronyssinus* (Der p), and investigate patterns of concomitant reactivity among different allergens to enhance the accuracy of HDM allergy diagnostics.

**Methods:**

A comprehensive analysis of 1520 patient test results in Lithuania from 2020 to 2022 was performed. Sensitization patterns to major (Der p 1, Der p 2, and Der p 23) and minor (Der p 5, Der p 7, and Der p 21) Der p allergen components were described using molecular‐based diagnostics. Additionally, we investigated sensitization to allergen components from other allergen sources, including tropomyosins (Der p 10, Per a 7, Pen m 1, Ani s 3, Blo t 10) and arginine kinases (Pen m 2, Bla g 9, Der p 20).

**Results:**

This study reveals a high prevalence of HDM sensitization in Lithuania ‐ 481 individuals (45.38% of the sensitized group) exhibited sensitization to at least one Der p allergen component. Importantly, within the sensitized group, 37.21% of patients were sensitized to Der p 5, Der p 7, or Der p 21 in addition to major allergenic components. Distinct sensitization patterns were observed across different age groups, indicating the influence of age‐related factors. Furthermore, we confirmed cross‐reactivity between Der p 5 and Blo t 5 as well as between Der p 21 and Blo t 21, emphasizing the clinical relevance of these associations. We also highlighted the complexity of sensitization patterns among tropomyosins and arginine kinases.

**Conclusion:**

This study provides valuable insights into HDM allergy sensitization profiles in Lithuania, emphasizing the importance of considering major and minor HDM allergen components for accurate diagnosis and management of HDM‐related allergic diseases. Differences between populations and age‐related factors impact sensitization patterns. Understanding concomitant reactivity among allergens, such as Der p 5 and Blo t 5, Der p 21 and Blo t 21, tropomyosins, and arginine kinases, is crucial for improving diagnostic strategies and developing targeted interventions for allergic individuals.

## 1 INTRODUCTION

Allergic diseases are among the most common chronic diseases worldwide and have a major impact on the quality of life of allergy sufferers. The prevalence of these diseases has increased worldwide to estimated 20%–30%. The crucial allergy associated health care costs and the economic burden of these diseases continue to increase.^1^, ^2^ Among the various allergenic sources, house dust mites (HDMs),—*Dermatophagoides pteronyssinus* (Der p), have emerged as significant triggers for respiratory allergic diseases as well as dermatitis. Up to 85% of allergen‐induced asthmatic patients are typically allergic to HDM despite of differences in geography, temperature and humidity.^3^, ^4^ HDM raises an important problem in the Lithuanian population, being one of the most prevalent allergens among sensitized patients. As many as 54.2% of patients sensitized to inhalant or food allergens were sensitized to HDM extracts.^5^ Despite vast number of studies available on HDM allergens, there are a number of areas that need to be further explored. Existing studies examining the frequency of sensitization exhibit a notable gap in terms of data and understanding concerning Eastern‐Southern European populations^6^, ^7^ Specifically, a large gap in knowledge of populational molecular allergen (MA) sensitization profiles can be observed in the Lithuanian population, with only one published study available, which primarily focused on determining the prevalence of sensitization to allergen extracts rather than immunomodulatory components.^5^ The importance of studying HDM allergens is underscored by its confirmation of previous longitudinal observations in the field,^8^ such as an in‐depth analysis of molecular sensitization profiles, uncovering the hierarchy in frequency, diagnostic, and clinical relevance of minor Der p allergens, categorization of molecules into high/mid/low frequency (group A, B, C) and importance of group A molecules.^8^ While group A molecules (Der p 1, Der p 2, and Der p 23) have long been recognized as primary Der p sensitization triggers^6^, ^8^, ^9^, ^10^ and have been at the epicenter of HDM epidemiological studies for many years, our knowledge on group B allergen (Der p 5, Der p 7, and Der p 21) prevalence is still lacking.^8^, ^10^, ^11^ As sensitization patterns of mite‐allergic patients are influenced by age, gender, heredity, and mite exposure^8^, ^10^ it is important to define Lithuanian population sensitization profiles to gain a better understanding of worldwide Der p sensitization trends.^12^, ^13^, ^14^, ^15^

The aim of this study was to assess the sensitization profiles to MA's derived from house dust mite (*Dermatophagoides pteronyssinus*) in the Lithuanian population. Specifically, we aimed to determine the frequency of genuine sensitization (primary, species‐specific sensitization) and cross‐sensitization (sensitization due to cross‐reactivity) to house dust mite among individuals with confirmed allergic sensitization.

## 2 METHODS

A retrospective study of 1520 anonymized patient test results was conducted in Lithuania. The study participant inclusion criteria consisted of a suspicion of atopic disease and routine screening of possible sIgE reactivity via ALEX^2^ macroarray test (MacroArray Diagnostics GmbH, Austria). The patient data were analyzed for the period spanning from 2020 to 2022.

The ALEX^2^ macroarray is a multiplex macroarray sIgE test that contains 295 antigens, including 117 extract allergens and 178 molecular components.^12^, ^13^ In this study, the focus was on analyzing sensitization to allergen components of *D*. *pteronyssinus*. The specific immunoglobulin E (sIgE) values for *D*. *pteronyssinus* Der p 1, Der p 2, Der p 5, Der p 7, Der p 10, Der p 11, Der p 20, Der p 21 and Der p 23 were determined. Furthermore, the study analyzed the frequency of genuine and cross‐sensitization to house dust mites (HDM) among all subjects with any confirmed allergic sensitization. Sensitization was defined by detecting a sIgE level of 0.3 kUA/L or higher.

In our study, the patient population was categorized into three distinct age groups: young children (under 12 years of age), adolescents (from 12 to 17 years of age), and adults (18 years of age and older). This division was implemented to examine the potential variations in allergic sensitization patterns across different stages of life. The patient population was also divided into groups based on gender.

The terms “monosensitized” and “polysensitized” in our study were used in the following way—we assumed monosensitization and polysensitization on a molecular level. As such, a monosensitized patient in our study was considered as a patient sensitized to 1 MA (not one allergen extract) and a polysensitized patient was considered as a patient sensitized to two or more different MA's regardless of allergen source. The retrospective study was approved by the Vilnius regional biomedical research ethics committee of Vilnius university and performed in accordance with all requirements.

Statistical analysis was carried out by SPSS 28.0 statistics program and Microsoft Excel. Baseline and demographic characteristics were summarized by standard descriptive summaries (medians and interquartile ranges for continuous variables and percentages for categorical variables). To compare differences, two‐sample Wilcoxon (or Kruskal‐Wallis) and χ^2^ tests were used for nonparametric continuous and categorical variables, respectively. In the statistical analysis, statistically significant data were considered when p value was <0.05. Additionally, UpSet plots were generated using the UpSetR package in R to visualize the intersection between more than 3 sets, providing insights into the relationships and overlaps within the sensitization patterns of the study participants.

## 3 RESULTS

### 3.1 Demographic characteristics of patients

The study comprised a total of 1520 patients with suspected atopic disease. The studied population consisted of 754 (49.61%) males and 766 (50.39%) females. 641 (42.17%) of patients were children under the age of 12, 124 (8.16%) individuals aged 12–17 years and 755 (49.67%) adults.

Sensitization to inhalant, food, or stinging insect venom allergens were detected in 1060 (69.74%) patients through the application of the ALEX2 macroarray.

Within the sensitized group of patients, 481 individuals (45.38% of the sensitized group) exhibited sensitization to at least one Der p allergen component.

Among HDM‐sensitized individuals, children under the age of 12 averaged at the age of 6.52 ± 2.45 years, 12 ‐ to 17‐year‐olds averaged at the age of 14.00 ± 1.65 years, and adults averaged at the age of 35.00 ± 10.01 years (Table 1).

**TABLE 1:** Demographic characteristics of patients.

Total patients (n=1520)			
		Frequency (%)	Mean age ± S.D
Gender	Males	754 (49.61%)	21.99 ± 16.832
	Females	766 (50.39%)	

Age groups	Sensitization status	Frequency (%)	Mean age ± S.D.
Sensitized patients (*n* = 1060; 69.74%)			
Children 0–11 years old	Non‐sensitized	207 (32.29%)	5.06 ± 2.586
	Sensitized	434 (67.71%)	6.52 ± 2.454
	Total	641	5.71 ± 2.628
Children 12–17 years old	Non‐sensitized	16 (12.90%)	13.93 ± 1.555
	Sensitized	108 (87.10%)	14.00 ± 1.650
	Total	124	13.97 ± 1.603
Adults	Non‐sensitized	237 (31.39%)	38.68 ± 10.60
	Sensitized	518 (68.61%)	35.00 ± 10.01
	Total	755	37.09 ± 10.50

	Frequency (% of sensitized patients in the group)		Mean age ± S.D.
HDM sensitized patients (*n* = 481, 45.38% of the sensitized group)			
Children 0–11 years old	195 (30.42%)		6.52 ± 2.454
Children 12–17 years old	62 (50.00%)		14 ± 1.65
Adults	224 (29.67%)		35 ± 10.01

### 3.2 Sensitization to any *D. pteronyssinus* molecular components

Statistically significant differences in sensitization frequency to any Der p allergen components among different age groups were observed (*p* = 0.026) among sensitized patients. Specifically, out of the patients who were sensitized to any of the tested allergens, the adolescent patient group (aged 12–17 years) displayed a higher frequency of sensitization to Der p allergens (50.00%) compared to little children (aged 0–11 years) and adults (30.42% and 29.67% respectively).

An evident disparity was observed in the frequency of sensitization to Der p allergen components between males (*n* = 279; 49.21%) and females (*n* = 201; 40.77%) (*p* = 0.006) when examining the frequency of Der p sensitization within the specific gender groups. Notably, males exhibited a significantly higher rate of sensitization to Der p allergen components compared with females.

### 3.3 Prevalence of *D*. *pteronyssinus* molecular components

350 (72.77%) children were positive for Der p 2 (either presented as molecular monosensitization or as combined sensitivity to other HDM allergens) and 289 (60.08%) for Der p 1. The third most prevalent component—Der p 23 was present in 56.97% (*n* = 274) of the studied sample. Based on these findings, we can conclude that these allergen components can be considered as major allergens in our population.

Sensitization to allergen Der р 21 was found in 107 (22.25%) patients either as molecular mono‐sensitivity, or combined sensitivity to other allergens, Der p 5 was present in 111 (23.08%) patients of the studied sample. These allergens can be considered as mid‐tier allergens (prevalence between 20% and 50%) in our population.

The remaining allergens (Der p allergens from groups 7, 10, 11 and 20) were verified as minor allergens (prevalence below 20%) in the whole study population (Table 2).

**TABLE 2:** Prevalence of Sensitization to Corresponding Der p Allergens in the Study Population, Stratified by Gender and Age.

	Prevalence of sensitized patients to corresponding allergen, *n* (%)	Prevalence of sensitization to corresponding allergen by age, *n* (%)				Prevalence of sensitization to corresponding allergen by gender, *n* (%)		
		Children 0–11 years old	Adolescents 12–17 years old	Adults	*p* ‐ value	Females	Males	*p* ‐ value
Der p 1	289 (60.08%)	130 (66.67%)	42 (67.74%)	117 (52.47%)	*P* **= 0.005**	117 (58.21%)	172 (61.65%)	*P* = 0.447
Der p 2	350 (72.77%)	128 (65.64%)	47 (75.81%)	174 (78.03%)	*P* **= 0.015**	142 (70.65%)	207 (74.19%)	*P* = 0.389
Der p 5	111 (23.08%)	38 (19.49%)	19 (30.65%)	54 (24.22%)	*P* = 0.168	46 (22.89%)	65 (23.30%)	*P* = 0.916
Der p 7	84 (17.46%)	32 (16.41%)	11 (17.74%)	41 (18.39%)	*P* = 0.868	25 (12.44%)	59 (21.15%)	*P* **= 0.013**
Der p 10	19 (3.95%)	10 (5.13%)	3 (4.84%)	6 (2.69%)	*P* = 0.412	4 (1.99%)	15 (5.38%)	*P* = 0.060
Der p 11	4 (0.83%)	0.00%	0.00%	4 (1.79%)	*P* = 0.098	1 (0.50%)	3 (1.08%)	*P* = 0.492
Der p 20	45 (9.36%)	20 (10.26%)	3 (4.84%)	22 (9.87%)	*P* = 0.418	18 (8.96%)	27 (9.68%)	*P* = 0.789
Der p 21	107 (22.25%)	43 (22.05%)	15 (24.19%)	49 (21.97%)	*P* = 0.928	44 (21.89%)	63 (22.58%)	*P* = 0.858
Der p 23	274 (56.96%)	103 (52.82%)	41 (66.13%)	129 (57.85%)	*P* = 0.169	111 (55.22%)	162 (58.06%)	*P* = 0.535

*Note*: Bold text indicates a statistically significant difference with a *p*‐value < 0.05.

Significant variations in the prevalence of sensitization to specific Der p allergen components were identified among different age groups in HDM‐sensitized patients. Notably, a lower prevalence of sensitization to Der p 1 was observed among adults, while a lower prevalence of sensitization to Der p 2 was observed among children aged less than 12 years compared to the other age groups (*p* = 0.005, *p* = 0.015, respectively). However, when considering other components of HDM allergens, the sensitization patterns did not show significant differences across the age groups (*p* > 0.05). (Table 2).

Variations in sensitization prevalence to specific Der p allergen components were observed in different gender groups. However, the only statistically significant difference was observed for Der p 7, with men exhibiting a higher sensitization frequency (*p* = 0.013). (Table 2).

### 3.4 Prevalence of individual molecular profiles in *D. pteronyssinus* positive patients

**FIGURE 1:**
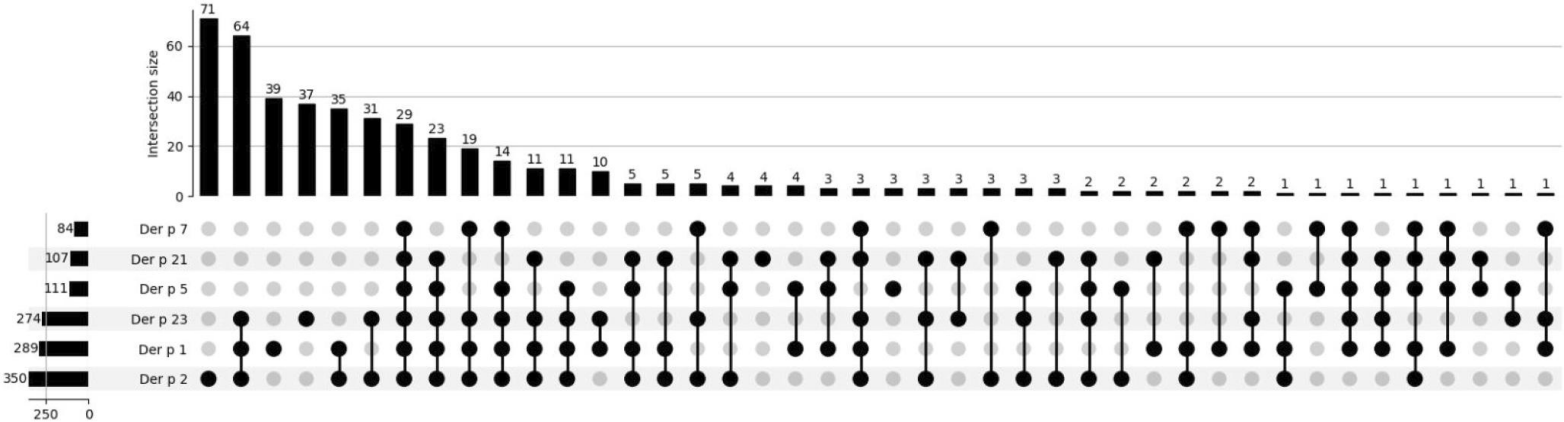
UpSet plot visualizing sensitization profiles for *Dermatophagoides pteronyssinus* specific allergen components.

Pleomorphic repertoire of molecular co‐sensitization was observed by evaluating all HDM allergen components (Der p 1, Der p 2, Der p 5, Der p 7, Der p 10, Der p 11, Der p 20, Der p 21 and Der p 23). Among HDM sensitized 481 children, a total of 72 distinct profiles were identified. As some of the Der p components exhibit cross‐reactivity with other species, we focused on identifying distinct sensitization profiles of the non—crossreactive components (including Der p 1, Der p 2, Der p 5, Der p 7, Der p 21, and Der p 23). A total of 42 distinct sensitization profiles where found among 466 patients (Figure 1). Molecular monosensitization to Der p 2 (*n* = 71; 15.24%) was the most frequent, followed by simultaneous sensitization to three specific components: Der p 1, Der p 2, and Der p 23 (*n* = 64; 13.73%). Molecular monosensitization to Der p 1 was present in 8.37% (*n* = 39), while monosensitization to Der p 23 was observed in 7.94% (*n* = 37) of studied individuals. The fifth most prevalent sensitization profile was the simultaneous sensitivity to Der p 1 and Der p 2 (*n* = 35; 7.51%). Followed by sensitization to Der p 2 and Der p 23 (*n* = 31; 6.65%) and a profile encompassing all six Der p‐specific molecular allergens (Der p 1, Der p 2, Der p 5, Der p 7, Der p 21, and Der p 23) which was detected in 6.22% of patients (Figure 1).

Out of 481 patients *n* = 37.21% (*n* = 179) were sensitized to at least one of the following MA's: Der p 5, Der p 7, and Der p 21. Among these patients, 65 individuals (13.51%) were sensitized to Der p 5, Der p 7, Der p 10, Der p 11, Der p 20, Der p 21, Der p 23 but not to Der p 1 and Der p 2. Notably, when excluding Der p 23, the number of patients sensitized to the remaining MA's (Der p 5, Der p 7, Der p 10, Der p 11, Der p 20, Der p 21) decreased to only 24 patients (4.88%). The forementioned prevalence includes cases where sensitization may occur due to cross‐reactions with MA's from other species. That being said, only 13 patients (2.70%) were sensitized to non‐major Der p specific allergens (Der p 5, Der p 7, Der p 21) without sensitization to Der p 1, Der p 2, or Der p 23.

### 3.5 Prevalence of individual molecular profiles in *D. pteronyssinus* positive patients among different age groups

Further analysis of specific sensitization profiles among different age groups (children aged 0–11 (*n* = 188), adolescents aged 12–17 (*n* = 62), and adults aged >18 years (*n* = 216)) was conducted. Age‐related differences were observed.

**FIGURE 2:**
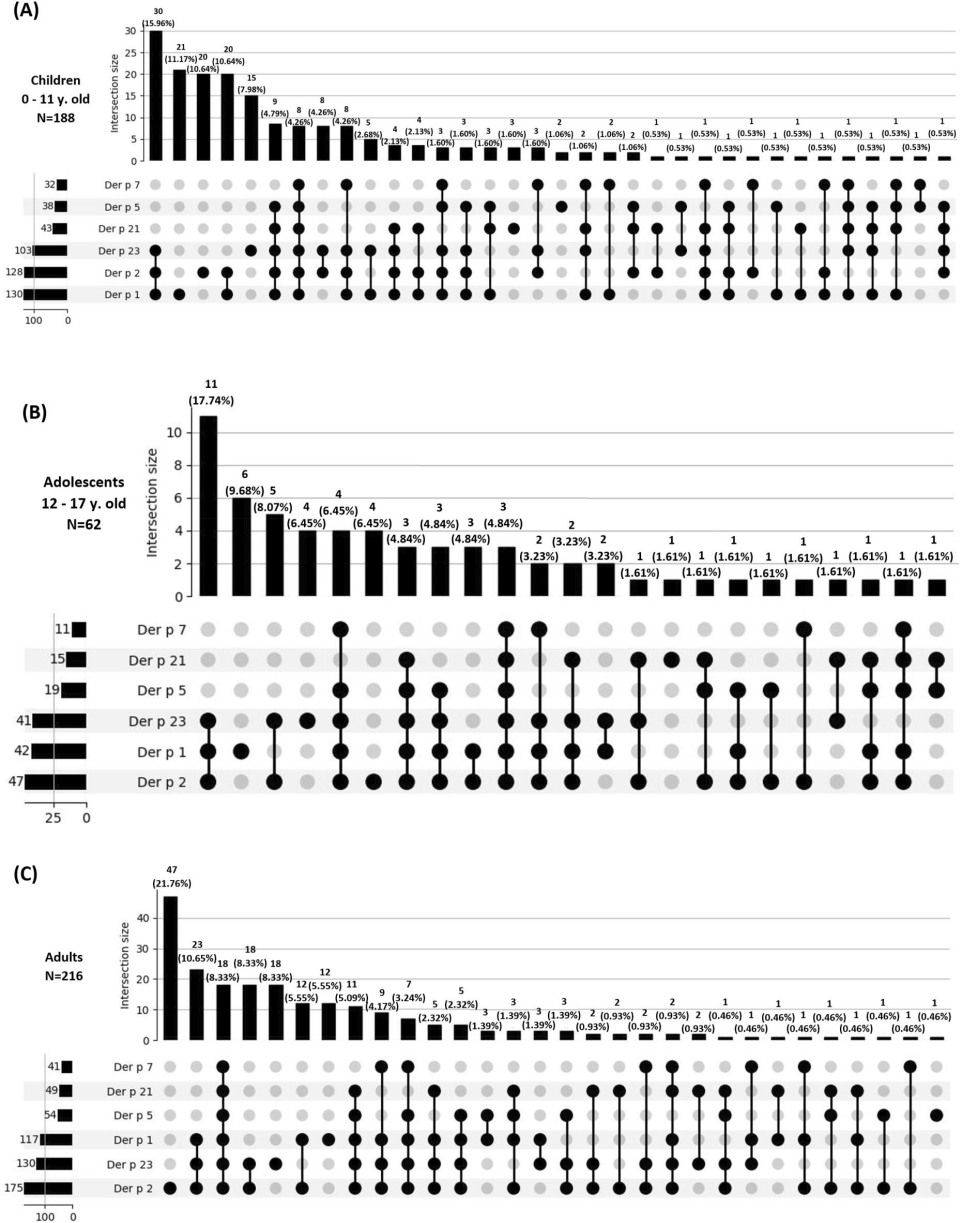
(A–C) UpSet plot visualizing sensitization profiles for *Dermatophagoides pteronyssinus* specific allergen components between different age groups.

Children aged 0–11 years were most frequently sensitized (15.96%) to three major Der p allergens (Der p 1, Der p 2, Der p 23). Followed by molecular mono‐sensitization to Der p 1 (11.17%), molecular mono‐sensitization to Der p 2 (10.64%) or co‐sensitization to Der p 1 and Der p 2 (10.64%). Sensitization to Der p 23 as a mono‐sensitization accounted for 7.98% of the cases, constituting the fifth most prevalent sensitization profile among children. These top five profiles accounted for 56.38% of sensitized children. Sensitization to mid‐tier and minor specific Der p allergen components (Der p 5, Der p 7, Der p 21) with or without co‐sensitization to Der p 1, Der p 2, and Der p 23 was observed in 36.70% of young children (Figure 2A).

Among adolescents aged 12–17 years, the most frequent sensitization profile (17.74%) also involved sensitization to the three major Der p allergens (Der p 1, Der p 2, Der p 23). Followed by molecular mono‐sensitization to Der p 1 (9.68%). Only these first two profiles were consistent with those in the children group. The third most prevalent profile among adolescents was molecular co‐sensitization to Der p 2 and Der p 23 (8.07%), followed by molecular mono‐sensitization to Der p 23 (7.69%). The fifth profile included sensitization to both major and rarer allergen components, namely Der p 1, Der p 2, Der p 23, Der p 5, and Der p 7. In the entire adolescent sample, sensitization to mid‐tier and minor specific Der p allergen components (Der p 5, Der p 7, Der p 21) was observed in 43.55% of patients (Figure 2B).

For adults aged 18 and above, the most frequent sensitization profile (21.76%) was molecular monosensitization to the Der p 2 MA. Followed by sensitization to Der p 1, Der p 2 and Der p 23 (10.65%). The third, fourth, and fifth most prevalent profiles were molecular co‐sensitization to Der p 1, Der p 2, Der p 23, Der p 5, Der p 7 and Der p 21; molecular co‐sensitization to Der p 2 and Der p 23; and molecular monosensitization to Der p 23, each accounting for 8.33% of patients. In the entire adult group, sensitization to mid‐tier and minor specific Der p allergen components was observed in 38.43% of patients (Figure 2C).

### 3.6 Concomitant reactivity among Der p 5 and Blo t 5/Der p 21 and Blo t 21

Study of potential sensitization relationships between Der p 21 and Blo t 21 MA's has been conducted. Among the patients sensitized to Der p allergens (*n* = 481), 20 individuals (4.16%) were also sensitized to Blo t 21. Further analysis revealed that all of these patients were also sensitized to Der p 21. This suggests a close association between sensitization to Der p 21 and Blo t 21; however, further studies are needed to prove molecular cross‐reactivity between these MA's.

Evaluation of possible sensitization relationships between Der p 5 and Blo t 5 MA's revealed that out of 48 patients sensitized to Blo t 5, 40 (83.33%) patients showed co‐sensitization to Der p 5. We observed 8 (16,67%) patients who displayed sensitization solely to Blo t 5 without concurrent sensitization to Der p 5.

Further analysis showed, that three individuals demonstrated exclusive sensitization to Blo t 5 without any possible cross‐reactions. In contrast, 5 patients exhibited co‐sensitization to Der p 21 alongside their sensitization to Blo t 5. Notably, these 5 patients with co‐sensitization to Der p 21 displayed remarkably high levels of specific IgE against Der p 21 (>40 kUA/l). In light of the substantial levels of specific IgE against Der p 21 exhibited by these 5 patients, one could speculate that there might be a possible association between their sensitization to Blo t 5 and their sensitization to Der p 21.

### 3.7 Concomitant reactivity among tropomyosin's (Der p 10, per a 7, Pen m 1, Ani s 3, Blo t 10)

**FIGURE 3:**
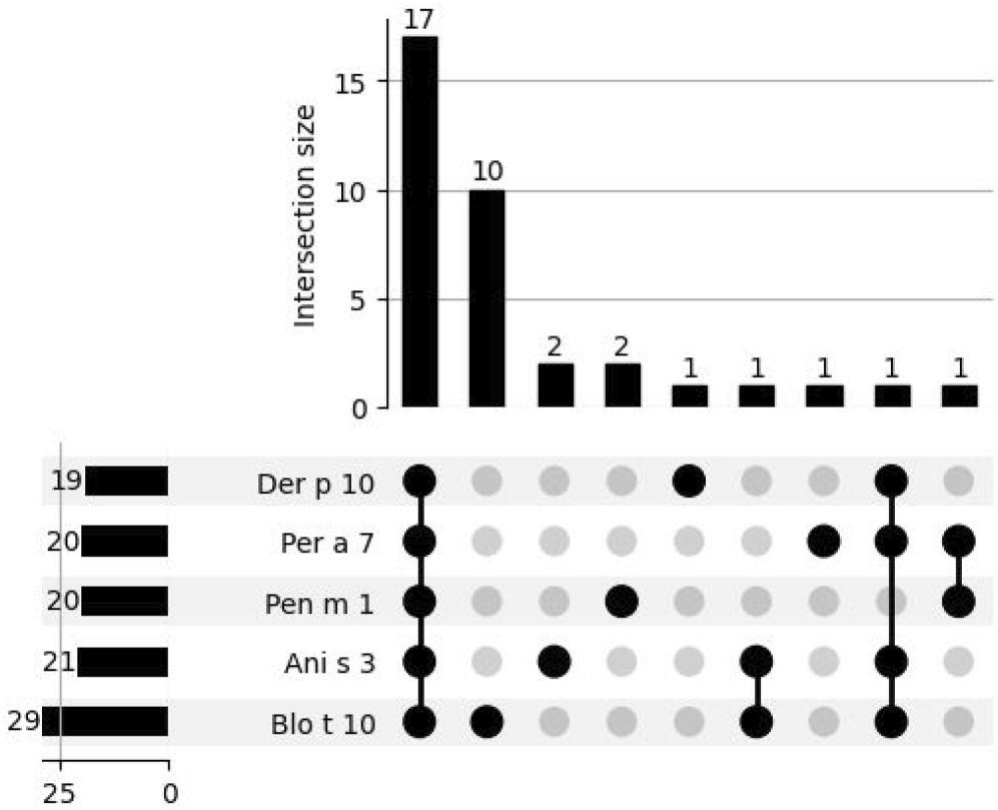
Sensitization profiles for tropomyosin‐specific allergen components: UpSet plot analysis.

Sensitization to tropomyosins was rarely observed within the studied population. Out of all sensitized patients, 3.40% (*n* = 36) exhibited sensitization to tropomyosins, and only 1.79% (*n* = 19) showed sensitization to Der p 10 MA. This accounted for 3.95% of all patients sensitized to HDM (Figure 3).

Only one patient displayed molecular monosensitization to solely Der p 10 tropomyosin. Although shrimp tropomyosin Pen m 1 is commonly regarded as one of the most important sensitizing invertebrate pan‐allergenic components, a significant proportion of patients sensitized to tropomyosins showed sensitization to other components besides Pen m 1 (*n* = 16, 44.44%) (Figure 3).

For many patients (*n* = 17; 47.22%) sensitized to tropomyosins, MA's profile encompassed all tropomyosin components included in the ALEX^2^ macroarray. However, it is noteworthy that monosensitization to Blo t 10 MA emerged as the second most prevalent sensitization profile among tropomyosin sensitization, with 27.78% (*n* = 10) exhibiting sensitization exclusively to Blo t 10 (Figure 3).

### 3.8 Concomitant reactivity among arginine kinase molecular allergens (Pen m 2, Bla g 9, Der p 20)

Sensitization to arginine kinase MA's was rarely observed within the studied population, with 4.53% (*n* = 48) exhibiting sensitization to arginine kinase out of all sensitized patients. Majority (81.25%) of them displayed sensitization to multiple arginine kinase MA's, indicating the presence of concomitant reactivity. The most common profile for arginine kinases, observed in 64.58% (*n* = 31), involved the simultaneous presence of all three tested components.

**FIGURE 4:**
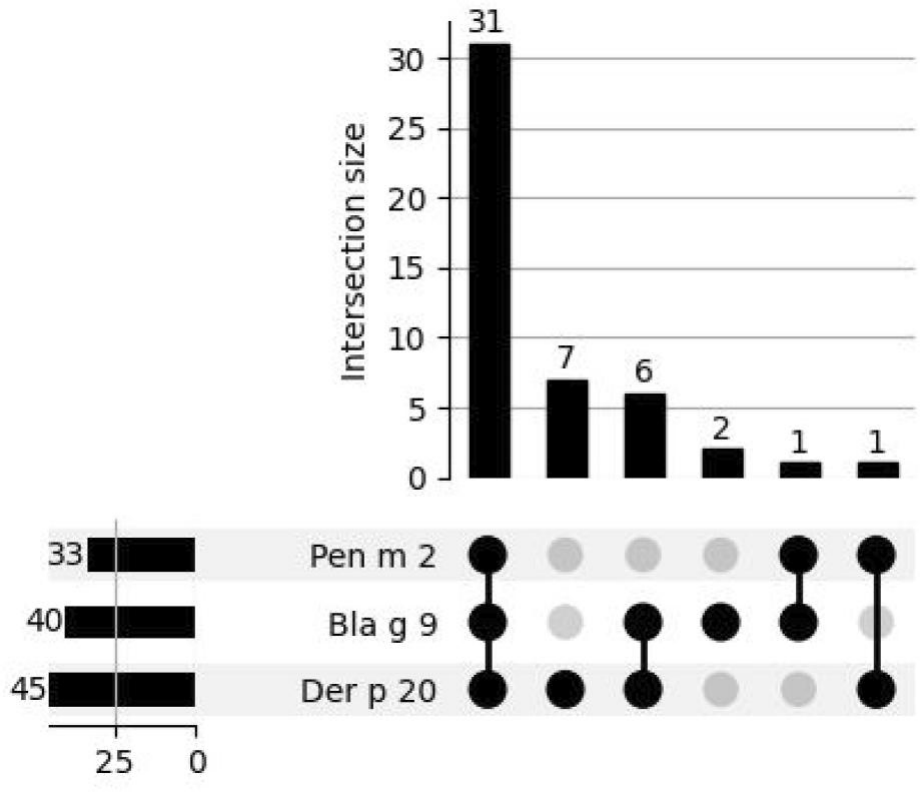
Sensitization profiles for arginine kinase‐specific allergen components: UpSet plot analysis.

The second most prevalent sensitization pattern for arginine kinases was Der p 20 molecular monosensitization in 14.58% (*n* = 7) of patients. Der p 20, the most common MA of arginine kinases, showed a higher sensitization frequency than Der p tropomyosin—Der p 10, affecting 45 (9.36%) versus 19 (3.95%) patients. Furthermore, the third most prevalent sensitization profile among arginine kinase MA's was the concomitant reactivity of Der p 20 and Bla g 9 observed in 6 (12.5%) patients (Figure 4).

## 4 DISCUSSION

Our study is the first to describe molecular sensitization patterns to HDM allergens in a Lithuanian population and to highlight the role played by age and gender, while simultaneously analyzing the phenomenon of concomitant reactivity among different allergens.

^18^, ^19^, ^20^Immediate hypersensitivity to indoor allergens increases the risk of asthma, allergic rhinitis, and atopic dermatitis. HDM sensitization is globally recognized as the leading cause of inhaled allergen‐triggered allergies. Sensitization prevalence varies worldwide, emphasizing the need for population‐specific studies.^14^, ^15^, ^16^ In Central Europe, Panzner et al.^17^ found mite‐specific molecule sensitization in 32.7% of patients, while in Poland, Ukleja‐Sokołowska et al.^18^ reported 59% sensitization to Der p allergens of sensitized patients and Rodinkova et al.^19^ discovered 27.00% HDM sensitization of general Ukraine population. In Lithuania, Der p sensitization was 45.38%, aligning with global findings that HDMs are pivotal allergens.

^24^, ^25^, ^26^Our study focused on analyzing sensitization to MA's of Der p and aimed to fill the gap in knowledge regarding sensitization patterns in the Lithuanian population. Among HDM‐sensitized patients 457 (95.01%) exhibited profiles involving major components (Der p 1, Der p 2, Der p 23). However, testing solely for these components would miss sensitization in 2.70% (only sensitized to Der p 5, Der p 7, Der p 21) and overlook 37.21% sensitized to them in addition to major allergens. Recognizing mid‐tier/minor components like Der p 5, Der p 7, Der p 21 is crucial for accurate diagnosis, considering their unique immunological properties.^20^, ^21^, ^22^ As previously described by Posa et al.^8^ sensitization to group B HDM MA's (Der p 5, Der p 7, and Der p 21) shows a significantly higher risk of mite related AR and asthma than unsensitized participants. A comprehensive understanding of both major and minor HDM allergen sensitization is vital for improved diagnosis and management of HDM‐related allergic diseases.

Muddaluru et al. analyzed molecular sensitization profiles to HDM MA's in 685 allergic individuals from various world regions.^6^ In their study, 75% of the European population showed sensitization to Der p 1, 86% to Der p 2%, and 73% to Der p 23. Our findings show statistically significantly (*p* < 0.001) lower sensitization rates (60.08% for Der p 1, 72.77% for Der p 2%, and 56.96% for Der p 23) than those described in the above study. This study also reported that 89% of respondents had sIgE to at least one of the group 1 or group 2 allergens,^6^ our findings indicated a similar percentage (86.49%). However, the percentage of European children with sIgE to any of the major allergens was slightly higher 92%^6^ vs. 95.01% our population. This suggests a trend of lower sensitization rates to Der p 1, Der p 2, or Der p 23 as individual components in our population compared to the rest of Europe, that might be explained by the higher prevalence of mono‐sensitization in our population compared to southern Europe (*p* = 0.042).^6^

We are reporting a 30.15% molecular mono‐sensitization rate to HDM components and 28.59% to any major HDM MA's. Rodinkova et al. reported 22.47% molecular mono‐sensitization rates to major allergens, with 12.52% sensitized to Der p 2, 6.33% to Der p 23%, and 3.62% to Der p 1^19^. Limão et al. reported 11.27% molecular mono‐sensitization rates to major allergens, 7.5% to Der p 2, 2.8% to Der p 23%, and 0.9% to Der p 1^28^, in Portugal.^23^ Mono‐sensitization to HDM MA's differs between populations. These disparities might be explained by differences in population characteristics, such as allergy comorbidities and geographical origin.

Koch et al. reported Der p 2 to be the most common molecular mono‐sensitization, followed by Der p 1^24^. These findings align with the results of our study. This study did not find any mono‐sensitization to Der p 5, Der p 7, Der p 11, or Der p 21 allergens, suggesting that these allergens may not be necessary in HDM allergen test panels.^24^ In contrast, our study identified 1.46% molecularly mono‐sensitized patients to either Der p 5 or Der p 21, highlighting the need to include these allergens in IVD diagnostic protocols for accurate diagnosis.

Pinheiro et al.^25^ suggested that Der p seven in addition to major HDM MA's is crucial for accurate diagnosis. While the clinical role of Der p seven is not yet fully understood, it is believed that hypersensitivity to Der p 7 can serve as a marker for asthma, as described by Curin et al.^26^ A significant proportion of Der p‐sensitized patients (17.46%) showed sensitization to this MA, even though we did not find any Der p 7 molecular mono‐sensitizations. These findings suggest that the diagnosis of HDM sensitization requires an expanded array of HDM MA's, encompassing both major and minor MA's. As new important HDM allergens continue to emerge, it is crucial to consider expanding the range of MA's used in routine patient diagnostics. For instance, the recently discovered allergen Der p 37, which has been linked to asthma, could become a candidate for inclusion in routine diagnostics. Patients sensitized to Der p 37 exhibited a more complex reactivity pattern, and the prevalence of sensitization to Der p 37 ranges from 19% to 28%.^27^, ^28^

Rodinkova et al. discovered that the most prevalent sensitization profile in the Ukrainian population was molecular mono‐sensitization to Der p 2 (12.52%), followed by simultaneous sensitivity to Der p 1 Der p 2 and Der p 23 (9.22%) and to Der p 1, Der p 2, Der p 21, Der p 23, Der p 5, and Der p 7 (7.72%).^19^ Our study also showed molecular mono‐sensitization to Der p 2 (*n* = 71, 15.24%) to be the most prevalent, followed by molecular co‐sensitization to Der p 1, Der p 2 and Der p 23 (*n* = 64, 13.73%). The third most prevalent sensitization profile in our study was molecular mono‐sensitization to Der p 1 (8.37%), which differed from Rodinkova et al. findings. Muddaluru et al. study reported sensitization to all 3 major Der p allergens (Der p 1, Der p 2 and Der p 23) to be the most common, while only 3.1% and 3.8% of their children recognized exclusively group 1 and group 2 allergens, respectively.^6^ Gonzalez‐Perez et al. reported sensitization to 6 specific molecules (23.65%) ‐ Der p 1, Der p 2, Der p 5, Der p 7, Der p 21, and Der p 23^29^ to be the most prevalent in Tenerife, Spain. This profile was the seventh most common sensitization profile in our population (6.22%). However, this frequency was identified in both moderate and severe asthmatics. Thus, keeping in mind Psoa et al.^8^ previous observations we could speculate that this profile is related to the onset of asthma and differences could be explained by our sample group having a wide range of allergy‐like symptoms.

We have observed a higher prevalence of monosensitization to Der p 1 among younger individuals in comparison to adults. Furthermore, we have noted that broader sensitization to Der p allergen components tends to be more pronounced in older patients. Our study confirms observations made by Psoa et al.^8^ with group B (Der p 5, Der p 7, and Der p 21) HMD MA's being prevalent in 15%–30% of our population. When comparing the expansion to these MA's with age, we notice significant difference in growing sensitization numbers to these MA's in adolescents versus children (Der p 5—from 19.49% to 30.65%, Der p 7—from 16.41% to 17.74%, Der p 21—from 22.05% to 24.19%). This suggests that age‐related factors, such as duration and intensity of exposure, immune maturation, and genetic predisposition, may influence the sensitization patterns to specific allergens.^7^, ^30^, ^31^

Our study examined the cross‐reactivity between Der p 5 and Blo t 5 as well as between Der p 21 and Blo t 21. Der p 5 and Blo t 5, a homologous allergen from *Blomia tropicalis*, share structural similarities and exhibit cross‐reactivity according to the literature.^32^, ^33^ The same can be said for group 21 allergen family members ‐ Der p 21 and Blo t 21.^33^, ^34^ This indicates that individuals sensitized to Der p 5 or Der p 21 may experience allergic reactions upon exposure to Blo t 5 or Blo t 21, respectively; however, additional studies using more sophisticated methods are needed to determine the prime sensitizer (Blo t or Der p). Our study results show substantial levels of specific IgE against Der p 21 exhibited by some patients sensitized only to Der p 5; one could speculate that there might be a possible association between their sensitization to group 5 MA's and group 21 MA's. This speculation can be supported by some literature which provides evidence supporting these associations.^34^, ^35^ It is important to note that additional factors, such as individual variations in immune responses and exposure patterns, may also contribute to the observed sensitization patterns.^34^ Further research is needed to elucidate the underlying molecular and immunological mechanisms driving group 5 and group 21 allergen cross‐reactivities and their clinical implications.

Tropomyosins, including Der p 10, Per a 7, Pen m 1, Ani s 3, and Blo t 10, are highly conserved proteins found in various allergen sources, such as dust mites, cockroaches, shellfish, and other arthropods. Due to their structural similarities, tropomyosins can exhibit cross‐reactivity, leading to allergic responses in individuals sensitized to these MA's.^36^, ^37^ Our study findings underscore the complexity of sensitization patterns associated with tropomyosins and highlight the presence of cross‐reactivity among different tropomyosin allergens. Within our study sample, sensitization to Blo t 10 was most prominent among studied tropomyosins. This was also observed by González‐Pérez, not only was Blo t 10 more frequently identified than Der p 10, but also selective Blo t 10 responses were detected.^38^ The prominence of sensitization to Blo t 10, along with the varied sensitization profiles observed within this group of allergens, suggests the need for further investigation into the specific molecular mechanisms underlying these sensitization patterns. Understanding the intricate sensitization patterns and cross‐reactivity among tropomyosin allergens is crucial for accurate diagnosis and appropriate management, and in the near future, a possible targeted allergen‐specific immunotherapy in patients with tropomyosin sensitization.^39^

We explored concomitant reactivity among arginine kinase MA's that have been identified as allergens in different arthropods, namely focusing on Pen m 2, Bla g 9 and Der p 20.^40^ Our findings demonstrate concomitant reactivity among these arginine kinase allergens, indicating shared immunological characteristics. The complexity of sensitization patterns associated with arginine kinase allergens is evident from our findings and other studies,^40^ highlighting the need for further research.

### 4.1 Limitations of the study

It is important to acknowledge certain limitations that should be considered when interpreting the results of this study. The cross‐sectional design inherently restricts our ability to establish causality or determine the time‐based sequence of events.

Secondly, our study focused exclusively on the Lithuanian population, limiting the generalizability of our findings to other geographical regions. However, as existing studies examining the frequency of sensitization to various allergens often exhibit a notable gap in terms of data and understanding of Eastern‐Southern European populations, and our study's inclusion of a sizable sample from the Lithuanian population adds to the diversity of existing research.

We focused primarily on the assessment of sensitization profiles and concomitant reactivity among specific allergens. Although these findings provide important insights into the immunological aspects of allergic diseases, further investigations are warranted to explore the clinical implications and the impact of these sensitization patterns on disease severity, treatment response, and long‐term outcomes.

## 5 CONCLUSION

In conclusion, we aimed to evaluate sensitization profiles to molecular allergens derived from Der p within the Lithuanian population and investigate concomitant reactivity among different allergens. Our findings contribute valuable insights to the field, enhancing the accuracy of allergy diagnostics and optimizing management strategies. Understanding sensitization patterns to major and minor HDM allergen components is crucial for improved diagnosis and management of HDM‐related allergic diseases. Differences in sensitization profiles were observed between populations, highlighting the influence of geographical and population‐specific factors. Age‐related factors were also found to impact sensitization patterns to specific allergens. The study confirmed the association between Der p 5 and Blo t 5, as well as Der p 21 and Blo t 21, indicating possible cross‐reactivity between these allergens. Additionally, concomitant reactivity was observed among tropomyosin allergens. Further research is needed to understand the underlying mechanisms and implications of these sensitization patterns.

## AUTHOR CONTRIBUTION

**Gabija Biliute**: Conceptualization (equal); Data curation (equal); Formal analysis (lead); Investigation (equal); Methodology (equal); Visualization (lead); Writing – original draft (lead); Writing – review & editing (equal). **Monika Miskinyte**: Conceptualization (equal); Data curation (equal); Formal analysis (supporting); Investigation (equal); Methodology (equal); Project administration (equal); Resources (equal); Supervision (equal); Validation (equal); Writing – review & editing (equal). **Asta Miskiniene**: Conceptualization (equal); Data curation (equal); Formal analysis (supporting); Investigation (equal); Methodology (equal); Project administration (lead); Supervision (equal); Writing – review & editing (equal). **Aukse Zinkeviciene**: Data curation (equal). **Violeta Kvedariene**: Data curation (equal); Project administration (equal); Supervision (equal); Writing – review & editing (equal).

## CONFLICT OF INTEREST STATEMENT

Authors Asta Miskiniene and Monika Miskinyte were employed by the company JSC "In Novum" ‐ distributors of ALEX (Allergy Xplorer) test. The remaining authors declare that the research was conducted in the absence of any commercial or financial relationships that could be construed as a potential conflict of interest.

## Data Availability

The data that support the findings of this study are available on request from the corresponding author. The data are not publicly available due to privacy or ethical restrictions.
